# The Kynurenine Pathway as a Potential Target for Neuropathic Pain Therapy Design: From Basic Research to Clinical Perspectives

**DOI:** 10.3390/ijms222011055

**Published:** 2021-10-13

**Authors:** Katarzyna Ciapała, Joanna Mika, Ewelina Rojewska

**Affiliations:** Department of Pain Pharmacology, Maj Institute of Pharmacology, Polish Academy of Sciences, 31-343 Krakow, Poland; kat.ciapala@gmail.com

**Keywords:** tryptophan, kynurenines, neuropathy, metabolic pathway, analgesia, kynurenic acid, 3-hydroxykynurenine, quinolinic acid, neuropathy

## Abstract

Accumulating evidence suggests the key role of the kynurenine pathway (KP) of the tryptophan metabolism in the pathogenesis of several diseases. Despite extensive research aimed at clarifying the mechanisms underlying the development and maintenance of neuropathic pain, the roles of KP metabolites in this process are still not fully known. Although the function of the peripheral KP has been known for several years, it has only recently been acknowledged that its metabolites within the central nervous system have remarkable consequences related to physiology and behavior. Both the products and metabolites of the KP are involved in the pathogenesis of pain conditions. Apart from the neuroactive properties of kynurenines, the KP regulates several neurotransmitter systems in direct or indirect ways. Some neuroactive metabolites are known to have neuroprotective properties (kynurenic acid, nicotinamide adenine dinucleotide cofactor), while others are toxic (3-hydroxykynurenine, quinolinic acid). Numerous animal models show that modulation of the KP may turn out to be a viable target for the treatment of diseases. Importantly, some compounds that affect KP enzymes are currently described to possess analgesic properties. Additionally, kynurenine metabolites may be useful for assessing response to therapy or as biomarkers in therapeutic monitoring. The following review describes the molecular site of action and changes in the levels of metabolites of the kynurenine pathway in the pathogenesis of various conditions, with a particular emphasis on their involvement in neuropathy. Moreover, the potential clinical implications of KP modulation in chronic pain therapy as well as the directions of new research initiatives are discussed.

## 1. Introduction

Pain is one of the most common symptoms that accompany many medical conditions. It acts as a part of the body’s defense system, as it induces reflexive retraction from the painful stimulus and tendencies to avoid that dangerous situation in the future; therefore, it has relevant evolutionary functions. The International Association for the Study of Pain (IASP) proposed the following to define the condition of pain: “Pain is an unpleasant sensory and emotional experience associated with actual or potential tissue damage or described in terms of such damage”. Nociceptive pain refers to pain clearly associated with tissue damage or inflammation [1], while nociplastic pain arises from impaired nociception despite no signs of potential or fatal tissue damage and no signs of damage or disease to the somatosensory nervous system [2]. This definition de facto includes pain complaints that occur despite the lack of nociception or neuropathy. Taking into consideration that the integration of sensations, emotions, and cognition influences the perception of pain, the term of psychogenic pain was also distinguished. This pain occurs without anatomic tissue injury and it is considered to be caused by psychological factors such as depression or anxiety [3]. Lesions or diseases of the somatosensory nervous system can result in the changed and disordered transmission of sensory signals to the spinal cord and brain, leading to the development of neuropathic pain [4]. This kind of pain is associated with conditions, such as spinal cord injury, stroke, multiple sclerosis, diabetes, cancer or can arise as a consequence of chemotherapy, or other inflammatory disorders [5]. As indicated by the research of van Hecke et al., 2014, the rates of morbidity have been divided into two groups: the first is chronic pain with neuropathic features (3–17% of the population), and the second is neuropathic pain associated with states such as painful diabetic peripheral neuropathy, post-herpetic neuralgia, trigeminal neuralgia, and glossopharyngeal neuralgia. Overall, according to this systematic review neuropathic pain affects approximately 7–10% of the general population [6]. People suffer from different sets of symptoms, including abnormal sensations (dysesthesia), pain caused by physiological, otherwise nonpainful stimuli of a continuous or paroxysmal nature (allodynia), or an exacerbated pain reaction to a noxious stimulus (hyperalgesia). Moreover, patients experience impressions, such as burning or coldness, tingling, numbness, or itching. Such manifestations can significantly decrease quality of life and negatively influence the physical, emotional, and social aspects of people’s functioning [7]. Furthermore, this condition is associated with other problems, such as loss of function, anxiety, depression, sleep disturbance, and impaired cognition [8]. Worse still, studies conducted by the IASP have shown that neuropathic pain is not relieved by standard analgesics; hence, it frequently becomes a condition with no prospects for significant improvement. The weaker effects of two main groups of analgesics (opioids and nonsteroidal anti-inflammatory drugs) can be observed in neuropathic pain management. For this reason, neuropathic pain therapy requires a more complex approach. Some data suggest that it is possible to achieve a significant (30–50%) reduction in pain in only 50% of patients [9]. Currently, substances with a strong recommendation and proposed as first-line drugs are tricyclic antidepressants (TCAs), pregabalin, gabapentin, and serotonin-noradrenaline reuptake inhibitors (SNRIs), whereas lidocaine, capsaicin patches, and tramadol are considered second-line treatments. Strong opioids, such as morphine and oxycodone, are suggested as third-line management, similar to botulinum toxin type A, which is recommended only for peripheral neuropathic pain or migraine [10,11]. Combined therapy is often used when treatment with one drug is not fully effective. Despite numerous clinical and experimental studies, the molecular mechanisms of the development and maintenance of neuropathic pain have not been fully clarified. Lesions, hypoxia, compression, inflammation, and chemical damage along the axons cause the degeneration of fibers and contribute to modifications in the expression of intracellular connections, which result in signal transmission disturbances [12]. As a consequence, an imbalance between excitatory and inhibitory signaling is present, which leads to the development of neuronal hypersensitivity in the central nervous system (CNS). Modified gene/protein expression in primary sensory neurons is believed to be the most important factor responsible for neuropathic pain progression. The alterations in ion channels, activation of immune cells, and release of glial mediators contribute to the sensitization of nociceptive pathways [13]. The shift in the microglial phenotype from a resting to an activated state appears to be especially significant since those cells, while activated, are a source of many pro-nociceptive compounds, such as chemokines, interleukins, growth factors, and other intracellular signaling molecules, which are responsible for the expansion of a neuroinflammatory cascade [14]. Overall, it is of great importance to understand the plastic changes within the nervous system after nerve injury because present pharmacotherapy focuses mostly on the alleviation of symptoms instead of treating the underlying pathophysiology. The development of an efficient, rational method for neuropathic pain relief is a major clinical issue. The lack of satisfactory therapeutic effects of third-level drugs of the analgesic ladder results in their increased dosage, which in turn leads to many adverse effects. Another major issue in the search for new forms of therapy is the necessity to perform preclinical studies using animal models. It is commonly known that there are differences and limitations between animal models of neuropathic pain and human neuropathy. However, a notable part of our understanding of the mechanisms of nociception comes from animal research whose findings have led to the emergence of useful therapeutic strategies. The investigation of the molecular mechanisms of neuropathic pain and testing of compounds with potential beneficial properties requires proper animal models, which is an irreplaceable link between in vitro studies using cell lines and clinical trials. The analysis of neuroimmune interactions in neuropathic pain continually presents new questions, the answers to which require thorough research. Since the kynurenine pathway is closely related to the immune system, questions about its role in nociception have also been raised. The aim of this review is to evaluate the role of tryptophan and its metabolites in the pathogenesis of neuropathic pain, taking into account the role of products and enzymes of the kynurenine pathway in other diseases. We also focused on which substrates and enzymes within the TRP (kynurenine) catabolic pathway could serve as potential therapeutic targets.

## 2. Kynurenine Pathway of Tryptophan Metabolism

**Scheme 1:**
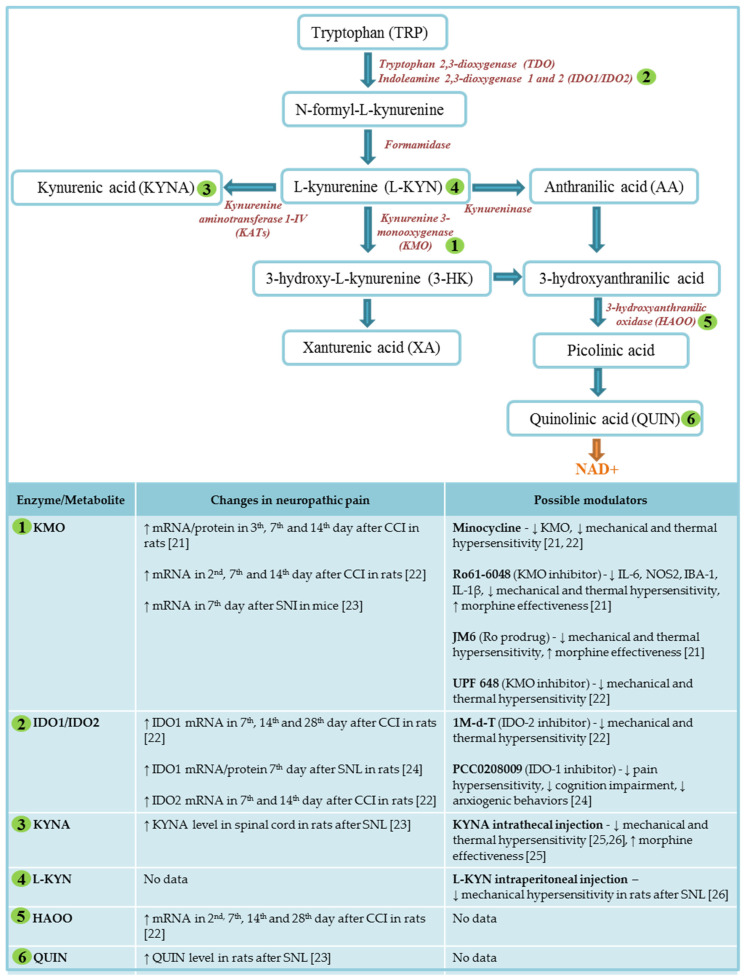
The kynurenine pathway of tryptophan metabolism and summary of differences in this pathway occurring in neuropathic pain with presentation of possible modulators [21,22,23,24,25,26].

Tryptophan (TRP) is an endogenous amino acid necessary not only for protein synthesis but also as a precursor to the synthesis of biologically active molecules, including nicotinamide adenine dinucleotide (NAD), nicotine acid, serotonin, and melatonin [15]. The kynurenine pathway (KP) and the better-known serotonin pathway are the two major routes of the TRP metabolism. The role of this amino acid in CNS functioning has been previously explored, mostly looking through the prism of serotonin, which is one of the best-known bioactive molecules. However, only 5% of dietary TRP is degraded to serotonin, whereas approximately 95% of TRP undergoes transformations through the KP, leading to the formation of kynurenines whose role is to regulate a plethora of biological processes, including the excitability of neurons, host–microbiome signaling, and the response of immune cells [16]. Tryptophan metabolism via the kynurenine pathway (Scheme 1) initiates with its conversion to N-formyl-L-kynurenine through three enzymes: tryptophan 2,3-dioxygenase (TDO) and indoleamine 2,3-dioxygenase 1 and 2 (IDO 1 and 2). N-formyl-L-kynurenine is rapidly converted to L-kynurenine, one of the major metabolites of the pathway. Subsequently, L-kynurenine can undergo three different transformations to kynurenic acid (KYNA) through the activities of the enzymes kynurenine aminotransferases (KATs) and 3-hydroxy-L-kynurenine (3-HK) with the participation of kynurenine 3-monooxygenase (KMO) and anthranilic acid (AA) due to kynureninase activity. In the next steps, 3-HK is modified to xanturenic acid (XA), while both 3-HK and anthranilic acid can be enzymatically converted to 3-hydroxyanthranilic acid. Next, 3-Hydroxyanthranilic acid is further metabolized by 3-hydroxyanthranilic oxidase (3-HAOO) to an unstable compound that is converted to picolinic acid and then nonenzymatically to quinolinic acid (QUIN), a precursor to the synthesis of NAD+ [15]. It is generally known that disorders of the CNS are often accompanied by disturbances in the TRP metabolism [17]. The products of the KP affect the endogenous regulation of neuronal excitability and mediate the regulation of the immune cell response, for example, via aryl hydrocarbon receptor (AhR) or G-protein coupled receptor 35 (GPR35). In recent years, the control of the kynurenine metabolism has been intensely studied, and many molecular sites and receptors have been proposed to be mediators of kynurenine action [18]. Among kynurenines, KYNA and QUIN are compounds that have gained great attention because they exert opposite properties—QUIN is a weak but specific agonist of the N-methyl-D-aspartate (NMDA) receptor and, hence, it bears neurotoxic potential, while KYNA is an endogenous nonselective antagonist of all subtypes of glutamatenergic receptors taking part in pain transmission, including NMDA, AMPA, and kainite [16]. Therefore, the KP was traditionally divided into “neuroprotective” KYNA and “neurotoxic” QUIN branches [15]. However, a number of multifarious properties of kynurenine metabolites have been revealed in the last decade, which also revealed that KYNA may contribute to the cognitive-depleting effects in patients with Alzheimer’s disease [19]. There are reports suggesting that this effect depends on the dose of KYNA; at low doses KYNA produces cognitive enhancement [20].

Moreover, both 3-hydroxykynureine and 3-hydroxyanthranilic acid have been related with both anti- and pro-oxidant properties [27]. Importantly, researchers focused on KP investigation have to face some challenges, not only technical or methodological but also theoretical. What we know about TRP degradation via the KP does not always agree with experimental results, raising many questions [28]. First, the kynurenine pathway varies across different species; some studies have reported that KMO and IDO-1 activity is higher in gerbils than in rats and that rat models do not fully reflect the KP course in humans [29,30,31]. Additionally, the cellular localization of kynurenine pathway enzymes and products is a matter of crucial importance. In vitro studies have revealed that the physical segregation of KP branches occurs in CNS: microglia/macrophages, which become activated in various neuroimmunological diseases, are equipped to form products of the “neurotoxic” arm of the KP, while astrocytes are responsible for the synthesis of neuroprotective KYNA [32,33]. This, however, requires further investigation since kynurenine pathway dynamics change substantially under nontypical conditions, and there is still a lack of appropriate antibodies against KP enzymes to further immunohistochemical studies [28]. Another important issue is the fact that peripheral kynurenines, indeed, affect brain function, although these processes are not entirely known. Apart from L-kynurenine, which is actively transported into the brain by the large neutral amino acid transporter, kynurenine-derived metabolites cross the blood–brain barrier (BBB) poorly [34]. Therefore, the concentrations of KP metabolites in the CNS are controlled mostly by local enzyme activity. These issues become complicated when, under pathological circumstances, inflammation induces changes in blood–brain barrier permeability. Undoubtedly, this condition significantly influences the different stages of the KP.

## 3. Neuroactive Metabolites of Kynurenine Pathway

### 3.1. L-kynurenine

L-kynurenine (L-KYN) is the key metabolite of the KP and the starting point for the synthesis of all the other metabolites in the pathway. Approximately 60–80% of L-KYN in the CNS is derived from peripheral sources, since under physiological conditions L-KYN is actively transported across the blood–brain barrier via the large neutral amino acid transporter [34,35]. Following systemic immune activation, all L-KYN levels in the brain come from the periphery, whereas, when inflammation is restricted to the brain, L-KYN can be formed locally from TRP. Electrophysiological experiments focused on L-KYN, 3-HK, 3-hydroxyanthranilic acid (3-HANA), and anthranilic acid have not revealed any direct influence of these compounds on neuronal activity. Systemic or intracerebral L-KYN injection evokes convulsions and influences blood pressure; however, these effects result from L-KYN conversion to its neuroactive metabolites [36,37,38]. Additionally, L-KYN itself displays various functions, including both pro- and antioxidant effects [27,39,40,41] and has been suggested to be an AhR agonist, albeit with an unknown downstream impact [42]. In glioblastoma cells, it has been shown that L-KYN action via AhR suppresses the antitumor immune response and promotes the survival of neoplastic cells [42]. It has also been hypothesized that the L-kynurenine-AhR axis may play a role in neurodegeneration and brain damage [43,44,45]. Increased concentrations of L-KYN in the blood and the CNS reflect the enhanced metabolism of TRP; thus, it is used as a biomarker for many neurological diseases.

### 3.2. Kynurenic Acid

The 4-Hydroxyquinoline-2-carboxylic acid was discovered by German chemist Justus von Liebig in 1853 in dog urine; hence, it was named kynurenic acid. At the end of the 20th century, KYNA was found to be present in the brain, and it was shown to be a nonselective antagonist of three types of ionotropic receptors for excitatory amino acids: the N-methyl-D-aspartate receptor (NMDAR), kainic acid receptor (KAR), and α-amino-2,3-dihydro-5-methyl-3-oxo-isoxazolpropionic acid (AMPA) receptor, with the strongest inhibitory effect on glutamatergic transmission resulting from blocking the strychnine-independent glycine part of the NMDA receptor complex [46]. Moreover, KYNA was suggested to regulate cholinergic transmission by acting as a noncompetitive antagonist of α-7 nicotinic receptors; however, this hypothesis has recently become controversial [47,48]. The neuroprotective effect of KYNA is mainly due to the inhibition of glutamate excitotoxicity through the antagonism of NMDAR. Importantly, approximately 80% of KYNA present in the brain is produced in glial cells, where it is not stored but released into the extracellular space by passive diffusion [34]. Carpenedo et al. have shown that the administration of even low concentrations of KYNA in the nanomolar range to the brain can reduce glutamate levels by 30–40%. This action involves blocking NMDA autoreceptors located on presynaptic nerve endings [49]. It was previously reported that a 2- or 3-fold increase in KYNA levels within the brain significantly diminishes post ischemic brain damage in in vivo models [50]. KYNA has also recently been shown to act as an agonist at GPR35, modulating cAMP production and preventing N-type Ca^2+^ channels in neurons and astrocytes [51]. Additionally, KYNA controls the immune response through its agonistic action at AhR, whose signaling appears to play an important role in inhibiting the release of cytokines in several types of immune cells, including macrophages [52]. KYNA levels are increased in the blood of patients suffering from multiple sclerosis [53], inflammatory bowel disease [54], and type 2 diabetes [55], while in the blood of patients with chronic schizophrenia [56], Alzheimer dementia [57], cluster headache [58], chronic migraine [59], and Parkinson’s disease [57] KYNA levels are decreased. Interestingly, the recent review pointed out that lover levels of KYNA accompany psychiatric disorders affecting cognitive domains, but higher levels of KYNA are observed in patients suffering from schizophrenia [20,60,61,62]. In people affected by Huntington’s disease, a reduction in KYNA in the cerebrospinal fluid and several brain regions was observed [60]. In patients suffering from rheumatoid arthritis, a positive correlation between serum KYNA and morning stiffness and pain was shown [61]. Additionally, it was observed that kynurenic acid exerts analgesic properties in an animal model of migraine, induced by dural inflammatory soup [62]. Therefore, it seems possible that pharmacological intervention affecting the production of KYNA—and at the same time the processes connected with the activity of excitatory amino acids in the CNS—may contribute to pain modulation.

### 3.3. 3-Hydroxy-L-kynurenine

Next, 3-Hydroxy-L-kynurenine (3-HK) is a metabolite of the KP, whose toxic effects are independent of the NMDA receptor, and is also a precursor to QUIN formation. Its neurotoxic properties are related to the production of free radicals [63], which in turn cause degeneration and apoptosis of neurons [39,64]. In addition, 3-HK generates the formation of superoxide and hydrogen peroxide, promoting copper-dependent oxidative damage to proteins [65]. In addition, it has recently been discovered that it has a negative impact on cell metabolism [66]. In contrast, some experimental studies have suggested that 3-HK displays antioxidant and scavenging properties because it acts as a two-electron donor; thus, it can restore the oxidative balance in the microenvironment [67]. Giles et al. proposed that the action of 3-HK depends on the redox status of the cell [68]. In the mammalian brain, 3-HK occurs in nanomolar amounts and is produced by activated macrophages and microglia. Stimulation of 3-HK has been observed in neurodegenerative diseases, such as hepatic encephalopathy, Huntington disease, Alzheimer’s disease, and dementia [69,70].

### 3.4. Quinolinic Acid

Pyridine 2,3-dicarboxylic acid, commonly known as a quinolinic acid (QUIN), was demonstrated to be an agonist of NMDA receptors, specifically subtypes containing the NR2A and NR2B subunits [71]. Schwarcz et al. discovered that QUIN induces selective neuronal injury and that its administration into the striatum is followed by pathological neurochemical changes [72]. Among NMDAR agonists, QUIN does not have a high-affinity uptake system for its removal from the synapse; therefore, it continuously acts on NMDARs, resulting in damage and massive calcium entry into neurons and astrocytes [73]. Briefly, the neurotoxic potential of QUIN includes various mechanisms, such as the stimulation of glutamate release, the inhibition of glutamate uptake by astrocytes, the production of reactive oxygen species, the reduction of endogenous antioxidants, as well as lipid peroxidation and cytoskeletal destabilization [71,74]. QUIN causes oxidative damage independent of its activity on the NMDA receptor through the formation of complexes with Fe^2+^, which mediates ROS production [75]. Importantly, it was also found that QUIN inhibits B monoamine oxidase (MAO-B) in human brain synaptosomal mitochondria and influences gluconeogenesis in the liver; thus, metabolic impairments also accompany its neurotoxicity [76]. Additionally, QUIN has been proven to regulate the inflammatory response. Intrastriatal QUIN injection induces the expression of tumor necrosis alpha (TNF-α) and interleukin-6 (IL-6) [73]. Within the CNS, QUIN is produced mostly by activated microglia and macrophages [71]. The quantity of QUIN in the brain and cerebrospinal fluid is approximately <100 nM; however, during pathological states, QUIN levels increase up to 500–1200 nM [77]. A growing body of research indicates that changes in QUIN levels have been observed in many neurodegenerative and immune-mediated diseases. Elevated QUIN levels are observed in Huntington disease [78], Alzheimer’s and Parkinson’s disease [79], depression [80], amyotrophic lateral sclerosis [81], human immunodeficiency virus (HIV) [82], and other conditions [83]. Importantly, a recent study involving over 17,000 patients with chronic pain recognized QUIN as the most commonly elevated biomarker and the authors suggested that its role in nociception is associated with peripheral NMDAR activation [84]. Ongoing research is focused on the use of NMDA receptor antagonists as a key therapeutic strategy in pain disorders [85].

## 4. Major Enzymes of Kynurenine Pathway

### 4.1. Indolamine, 2,3-dioxygenase-1 and 2

Indolamine, 2,3-dioxygenase-1 (IDO1) and IDO2 are two related, initial rate-limiting enzymes (along with the unrelated enzyme TDO) in the KP and have aroused great interest with regard to inducing cancer immunosuppression, tumor development, and metastasis and neovascularization [86,87]. Although IDO1 and IDO2 share a high level of sequence identity, they exert notably different functions and kinetics. It was found that IDO1 is the high affinity enzyme with KM (Trp) = ~5 µM and IDO2 is the low affinity enzyme with KM (Trp) = ~100 µM, and IDO2 can catalyze the reaction even when Trp > 200 µM [88]. Moreover, it was suggested that IDO1 mediates T cell suppressive effects while IDO2 works directly in B cells as a proinflammatory mediator [89]. IDO1 is expressed in various immune and nonimmune tissues, whereas IDO2 is expressed mostly by antigen-presenting cells (APCs) of the immune system, including dendritic cells (DCs) and B cells [90]. However, studies on primary microglia and astrocyte cultures have proved that the IDO2 enzyme is also derived from microglial and macrophage cells [22,91,92]. IDOs participate in the natural defense against various pathogens and have an immunosuppressive function because of their ability to limit T cell function and evoke mechanisms of immune tolerance. The effects of IDO activation are mediated not only via INF-y and the reduced availability of tryptophan, but also by downstream KP metabolites. For example, L-KYN and KYNA are able to activate the AhR, which in turn induces increased expression of IDO and TDO, creating a positive feedback loop [91,93,94]. Currently, many studies have described changes in IDO1 and/or IDO2 in many pathological conditions, such as amyotrophic lateral sclerosis, autoimmune diseases, Huntington disease, Alzheimer’s disease, Parkinson’s disease, cerebral ischemia, multiple sclerosis, as well as in tumors [91,92,93,94,95,96,97,98,99]. Many researchers have suggested that IDO2 could be an effective therapeutic target to treat inflammatory autoimmunity, while IDO1 has recently been under intense research focused on cancer immunotherapy. A few IDO1 inhibitors have been reported, some of which, including epacadostat, BMS986205, indoximod, and PF-06840003, among others, have advanced into clinical trials for cancer treatment [100].

### 4.2. Kynurenine-3-monooxygenase

The kynurenine-3-monooxygenase enzyme belongs to the NADPH-dependent flavin monooxygenase family [101]. Tissue distribution studies have shown that KMO in mammals is highly expressed within the liver and kidneys, while very low levels of this enzyme have also been detected in the CNS [102]. Macrophages, microglia, monocytes, and endothelial cells are mostly responsible for KMO synthesis. This enzyme converts L-KYN to 3-HK and, therefore, is responsible for QUIN formation. KMO is induced by IFN-1β, IFN-6, TNF-α, and oxygen molecule (O_2_), but inhibited by IL-4, IL-10, SOD, and by NADH which creates a negative feedback loop [103]. KMO expression levels are upregulated by proinflammatory cytokines, and an increased KMO metabolism results in the creation of compounds that activate glutamate receptors and elevate oxidative stress, which has fundamental consequences for CNS functioning. KMO plays a significant role in the regulation of peripheral inflammation [104] and central nervous system diseases [105]. In innate physiology, the KP participates in the regulation of early brain development [106]. It has been proven that the inhibition of KMO reduces the production of the “neurotoxic” branch of the KP and shifts the balance to the formation of KYNA. Therefore, KMO has become a very attractive drug target in many neurological diseases. However, KMO inhibitors have their limitations, mostly associated with poor blood–brain barrier permeability [107]. Over the years, many potent KMO inhibitors have been designed and confirmed to be beneficial in a variety of disease models. In the latest study, Zhang and colleagues created a new brain-permeable inhibitor of KMO [108]. Additionally, our previous study showed that at different time points after sciatic nerve injury (i.e., on days 3, 7, and 14) mRNA and *Kmo* protein levels in the spinal cord and dorsal root ganglia (DRG) increased. In 2014, we showed that the pharmacological inhibition of KMO weakened pain features and enhanced morphine effectiveness in a neuropathic pain model. We observed that chronic intraperitoneal or intrathecal administration of minocycline decreased pain and caused similar reductions in KMO mRNA levels in the spinal cord and the DRG. The inhibition of KMO has a therapeutic effect in several preclinical models of neuropathology, including neuropathic pain [21].

### 4.3. Kynurenine aminotransferases

Biologically active as homodimers, kynurenine aminotransferase (KAT) enzymes belong to the family of PLP-dependent enzymes and are involved in the production of neuroactive KYNA through the irreversible transamination of L-KYN [109]. KAT is distributed unevenly in the brain, with its highest activity observed in rhinencephalon and its lowest activity in the cerebellum, where it is mainly located in the cytoplasm of astrocytes and where only negligible amounts are present in nerve cells [110]. These relative proportions of the distribution of KAT are, however, different in the spinal cord. Almost the same amount of the enzyme is found both in glial cells and in neurons, with the enzyme concentrating in small grains bound to the cell membrane. Four KATs, I, II and III IV, have been identified in the mammalian brain [111]. KAT I, KAT II, and KAT III take part in the synthesis of KYNA and are characterized by different catalytic properties. It is likely that KAT II and III operate under physiological conditions, while KAT I may be of particular importance in pathological conditions, such as microglial activation. Since KYNA in the brain is mostly produced by KAT II, this enzyme is an important target for pharmacological modulation. [111,112]. However, the turnover kinetics of KAT II and KYNA, which could influence the duration of the pharmacological effect, have not been described thus far.

### 4.4. Kynureninase

Kynureninase (KYNU) is a pyridoxal phosphate-dependent enzyme; attenuation of its activity in vitamin B6 deficiency leads to the conversion of 3-hydroxykynurenine and L-KYN to 3-HAA and AA, respectively [27]. KYNU is an enzyme necessary for the biosynthesis of nicotinamide nucleotides [113] and picolinic acid and enhances the synthesis of nitric oxide [27]. There is evidence that the stimulation of kynureninase activity is crucial in inflammatory processes. KYNU has been shown to increase in cerebral and systemic inflammatory conditions [114]. Moreover, IFN-gamma has been shown to elicit kynureninase activity in murine macrophages [113]. Thus far, a role for KYNU has been demonstrated in such chronic inflammatory skin diseases as psoriasis and atopic dermatitis [115]. The role of this enzyme in general human immunology has not been extensively studied. KYNU is expressed in almost all body organs and is involved in various inflammatory and cardiovascular diseases [116]. Recently, its role in several types of cancers has been explored, especially in the development of breast tumors [117].

## 5. Kynurenine Pathway in Neuropathic Pain

**Scheme 2:**
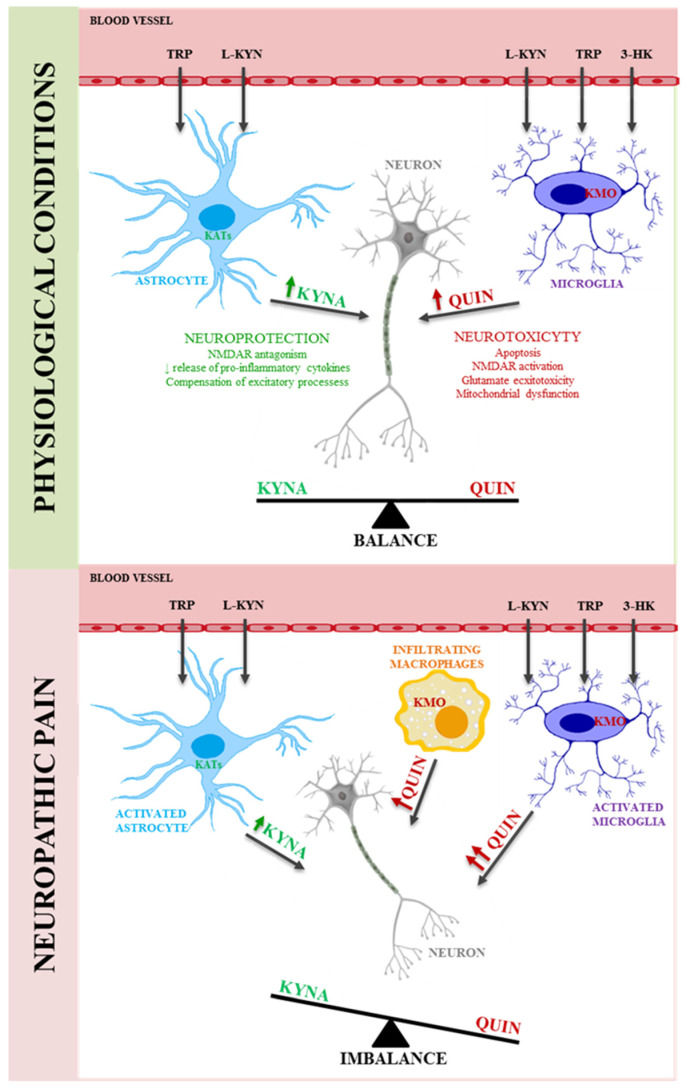
The changes in kynurenine pathway in neuropathic pain within the central nervous system. Under physiological conditions (**top panel**) there is a balance between neuroprotective KYNA produced by astrocytes and neurotoxic QUIN produced by microglia. In neuropathic pain (**bottom panel**), activation of microglia and infiltration of peripheral macrophages occur; therefore, as a consequence, the balance between KYNA and QUIN is disturbed.

A growing body of evidence indicates that sustained neuroinflammation contributes to pathological changes in the plasticity of sensory neurons, which, in turn, provokes chronic pain. Injuries and diseases that affect the nervous system can induce the activation of immune cells, alterations in capillary permeability, and the infiltration of peripheral blood cells. Taking into consideration the KP involvement in a plethora of physiological and pathological processes, and the fact that the source of its metabolites within the CNS are mainly glial cells, it becomes clear that glial cells should be the first target in order to define the role of kynurenines in neuropathic pain. First, Pineda-Farias et al. showed that intraperitoneal administration of L-kynurenine (50–200 mg/kg) and probenecid (100 mg/kg) diminished tactile hypersensitivity in rats exposed to spinal nerve ligation (SNL), which was associated with increased KYNA levels in the spinal cord [26]. An extensive screening study using DNA microarray revealed that one out of the genes whose expression was altered by chronic constriction injury of the sciatic nerve (CCI) in rats was *Kmo* [118]. Interestingly, the CCI-elevated mRNA level of *Kmo* was diminished in rats after chronic treatment with minocycline (a microglia/macrophage inhibitor). In the next study, it was proven that at different time points after a sciatic nerve injury, 2, 7, and 14 days after surgery, the mRNA and protein levels of KMO increased in the spinal cord and DRG. Additionally, these changes correlated with increased levels of microglial markers (CD40 and IBA-1) and astrocyte markers (GFAP). Moreover, the results showed that repeated intraperitoneal or intrathecal administrations of minocycline decreased the post injury elevated levels of *Kmo* mRNA in both examined structures, which was accompanied by reduced hypersensitivity to pain stimuli. Using primary glial cell cultures, it was confirmed that minocycline inhibited the LPS-stimulated increase in KMO mRNA in microglia but not in astroglia. The next stage explored the potential for the KMO inhibitors Ro61–6048 and JM6 to reduce neuropathic pain symptoms. Indeed, both substances reduced the mechanical and thermal hypersensitivity evoked by nerve injury, and a biochemical analysis indicated that Ro61–6048 reduced the levels of CD40/IBA1, IL-1β, IL-6, and NOS2 mRNA and/or proteins in both the spinal cord and DRG [21]. Recently, another study provided the first evidence that IDO1/2, KMO, and HAOO but not TDO elevated mRNA levels in the spinal cord, as measured on day 7 after the sciatic nerve injury in a rat model, parallel to C1q-positive cell activation. Extended pharmacological studies provided evidence that the repeated intraperitoneal administration of minocycline not only attenuated tactile and thermal hypersensitivity but also diminished the levels of IDO2 and KMO mRNA levels. Further pharmacological investigation confirmed that IDO2 and KMO enzymes take part in the development of neuropathic pain, in that it was observed that the repeated administration of IDO2 (1-M-d-T) and KMO (UPF 648) inhibitors diminished the development of hypersensitivity as measured on days 2 and 7 [22]. Using a spared sciatic nerve injury (SNI) model of neuropathic pain in mice, increased levels of Il-1β and *Kmo* mRNA in the contralateral side of the brain were found. The injury-induced increase in KMO mRNA was associated with the increased KMO protein and elevated QUIN and reduced KYNA levels in the contralateral hippocampus. The blockade of brain IL-1 signaling after SNI prevented the increase in KMO mRNA and depression-like behavior measured by the forced swim test, but had no effect on mechanical allodynia [23]. The importance of the IL-1β signaling pathway in the spinal cord has also been suggested to be crucial for the development of peripheral, IDO-1-mediated, injury-induced mechanical allodynia and depression-like behavior [119]. It has been demonstrated that the IDO1 inhibitor, PCC0208009, diminished pain hypersensitivity and cognition impairment in rats exposed to spinal nerve ligation [24]. Moreover, it has been shown that the intrathecal administration of KYNA reduced mechanical and thermal hypersensitivity in CCI-exposed mice. GPR35, expressed within the CNS in both neuronal and nonneuronal cells, including glia, has been suggested to be responsible for this effect [25]. The analgesic action of KYNA via GPR35 is possible due to modulating hyperpolarization-activated cyclic nucleotide-gated (HCN) channels [120]. These results correspond with previous reports showing that in rat neuropathic pain models, KYNA administered intrathecally reversed tactile hypersensitivity [26]. Importantly, the intrathecal administration of KYNA not only diminishes mechanical and thermal hypersensitivity but also potentiates the analgesic properties of morphine; however, the clarification of this phenomenon requires further study. Interestingly, the KMO blockade by Ro61–6048 and JM6 enhanced morphine effectiveness, probably due to decreases in IL-1β and IL-6, which are known to exert opposing effects on morphine efficacy [21]. Overall, under neuropathic pain, due to the microglia/macrophages activation there is an imbalance between neuroprotective KYNA and neurotoxic QUIN production (Scheme 2) which might be relevant in designing new pharmacotherapy for neuropathic pain.

## 6. Therapeutic Potential of the Kynurenine Pathway in the Treatment of Neuropathic Pain

The KP seems to be an important mediator in neuropathic pain pathology. It is possible that the modulation of its activity can be used in planning new treatment strategies. The mutual dependence between the mechanism of neuropathic pain and the activity of the kynurenine pathway will hopefully allow us to find a new mechanism of changes accompanying injury and create a possibility for using this knowledge in prophylaxis or therapeutic treatment. Management of neuropathic pain is consistently an enormous challenge for both health providers and patients. People affected by this kind of pain suffer from persistent symptoms that significantly reduce their quality of life, while doctors may propose treatment with a substance that gives only a partial improvement. In the context of the role of the kynurenine pathway in neuropathy, several points of interest in terms of pharmacological intervention can be distinguished. Most of them rely on manipulating the levels of neuroactive metabolites of this pathway. The first option is to use KMO inhibitors, since this enzyme initiates a cascade of transformations that result in the synthesis of the neurotoxin QUIN. This possibility is interesting because the results from basic, preclinical research indicate that these inhibitors indeed reduce hypersensitivity to mechanical and thermal stimuli. Moreover, from the therapeutic perspective, it is also significant that the inhibitors of KMO studied thus far intensify the analgesic effect of morphine. It is worth remembering that QUIN is also formed from anthranilic acid (Scheme 1); thus, the simultaneous use of an inhibitor of this enzyme could influence the analgesic effects of morphine. The direct precursor of QUIN is 3-hydroxyanthranilic acid, the modulation of which seems to be an interesting solution; however, the potential side effects should be taken into account, such as a decrease in the production of neuroprotective cinnabaric acid [121], which could be counterproductive.

Among the enzymes of the kynurenine pathway, IDO2 is an interesting target for therapeutic manipulation: its inhibition caused a decrease in hypersensitivity to mechanical and thermal stimuli after ligation of the sciatic nerve in an animal model of neuropathy. An extremely interesting compound is also KYNA, whose anticonvulsant and neuroprotective properties have previously been confirmed in numerous studies. There is a chance that pharmacological inhibition of QUIN production would result in a shift of the metabolic balance of the KP toward its neuroprotective branch (i.e., an increase in the synthesis of KYNA). It is worth noting that KYNA in combination with plasma proteins becomes practically impermeable to the BBB [34]. However, recently, there have been attempts to create nanoparticles that allow therapeutic amounts of kynurenic acid to penetrate the blood–brain barrier. The results of these experiments are very promising, showing that KYNA encapsulated in nanoparticles and administered peripherally to animals penetrates the blood–brain barrier and causes electrophysiological changes within the CNS [122]. Designing compounds comparable to KYNA in terms of chemical structure and biological activity remains a tremendous challenge. Several analogs of KYNA have already been synthesized: (2-(2-N,N-dimethylaminoethylamine-1-carbonyl)-1H-quinolin-4-one hydrochloride; KYNA-A1; 5,7-diBr-KYNA; 5,7-diCl-KYNA; 5-I,7-Cl-KYNA; and L-689,560), and their physical, chemical, and pharmacological properties are constantly being investigated. The recently discovered KYNA- or L-KYN-containing peptides are very promising in terms of pharmacological intervention. Kynurenine-containing peptides that influence opioid receptor activity have been previously isolated from natural organisms. Szűcs et al. designed and synthesized novel opioid peptide analogs combining L-KYN or KYNA in place of native amino acids. Importantly, this new, L-KYN-containing peptide displays a strong antinociceptive effect in in vivo tests [123]. It is worth mentioning because of the impact on the serotonin metabolism; the kynurenine pathway was already suggested to be important in migraine attack and under migraine treatment [124,125]. The crosstalk between the kynurenine pathway and tetrahydrobiopterin (BH4) pathway has been recently raised as a very important factor in nociception processes. Recently, XA was recognized as an endogenous inhibitor of the BH4 metabolism which raises the possibility that XA can potentially modulate the pathological overproduction of BH4 reported in chronic pain hypersensitivity animal models [126]. Given the dynamically developing research on the role and effect of KP modulators, the possibility for their clinical use in the treatment of various CNS disorders is arousing increasing interest. Perhaps, in the future, these compounds may also be among the pathways supporting the treatment of neuropathic pain.

## 7. Conclusions

In this review, we highlight emerging evidence of kynurenine pathway involvement in the development of neuropathic pain and other conditions and KP modulation as a potential strategy for the management of this kind of pain. An increasing number of studies showing that kynurenine and its metabolites can directly or indirectly influence and control various classical neurotransmitter systems has broadened our knowledge and aroused greater interest in the role of the KP in nociception processes. In addition, as research has shown, pain, stress, and infections modulate the metabolism of the kynurenine pathway and cause serious changes in well-being. In the era of dynamically developing research on the kynurenine pathway, increasing interest has been aroused by the possibility for the clinical use of its metabolites, both in terms of the prediction of treatment effects as well as new possibilities in the treatment of CNS disorders. Therefore, there is a need to continue to expand research in this area, which in the future may allow for more effective therapy that is less burdensome for people affected by CNS disorders, reduce costs and, above all, improve the patient’s quality of life. In the future, research should be directed towards identifying changes in tryptophan metabolism in people suffering from neuropathic pain of various etiologies. Moreover, keeping in mind that this kind of pain has a significantly higher prevalence among females compared with males, the gender differences are extremely important aspects, and relatively not yet explored in the matter of kynurenine pathway disturbances. Most likely, in the near future, new, thus far unknown, physiological and pathological properties of kynurenines will be discovered and interest in developing drugs based on modifications of the KP will increase. Complete knowledge of the KP will be critical to one of the major advances in medical research, by preventing disease rather than treating symptoms.

## Data Availability

Not applicable.

## Abbreviations

1-M-d-T	1-Methyl-D-tryptophan
3-HANA	3-hydroxyanthranilic acid
3-HAOO	3-hydroxyanthranilic oxidase
3-HK-3	hydroxykinurenine
AA	anthranilic acid
AhR	aryl hydrocarbon receptor
AMPA	α-amino-2,3-dihydro-5-methyl-3-oxo-isoxazolpropionic acid
APCs	antigen-presenting cells
BBB	blood–brain barrier
BH4	tetrahydrobiopterin
C1q	complement component 1q
CCI	chronic constriction injury
CD40	cluster of differentiation 40
CNS	central nervous system
DCs	dendritic cells
DRG	dorsal root ganglia
GFAP	glial fibrillary acidic protein
GPR35	G-protein coupled receptor 35
HCN channels	hyperpolarization-activated cyclic nucleotide–gated channels
HIV	human immunodeficiency virus
IASP	The International Association for the Study of Pain
IBA-1	allograft inflammatory factor 1
IDO	indoleamine 2,3-dioxygenase
IFN-1β	interferon beta -1
IFN-6	interferon-6
IFN-gamma	interferon gamma
IL-1	interleukin 1
IL-10	interleukin-10
IL-1β	interleukin 1 beta
IL-4	interleukin-4
IL-6	interleukin-6
KAR	kainic acid receptor
KATs	kynurenine aminotransferases
KM	Michaelis constant
KMO	kynurenine-3-monooxygenase
KP	kynurenine pathway
KYNA	kynureinc acid
KYNU	kynureninase
L-KYN	L-kynurenine
L-KYN	L-kynurenine
MAO-B	B monoamine oxidase
NAD	nicotinamide adenine dinucleotide
NMDA	N-methyl-D-aspartate
NOS2	Nitric oxide synthase 2
QUIN	quinolinic acid
ROS	reactive oxygen species
SC	spinal cord
SNI	sciatic nerve injury
SNL	spinal nerve ligation
SNRIs	serotonin-noradrenaline reuptake inhibitors
SOD	superoxide dismutase
TCAs	tricyclic antidepressants
TDO	tryptophan 2,3-dioxygenase
TNF-α	tumor necrosis alpha
TRP	tryptophan
UPF648	(1S,2S)-2-(3,4-Dichlorobenzoyl)cyclopropanecarboxylic acid
XA	xanturenic acid
